# Anti-N-methyl-D-aspartate receptor encephalitis associated with chronic myelogenous leukemia, causality or coincidence? A case report

**DOI:** 10.1186/s12883-022-02675-5

**Published:** 2022-04-23

**Authors:** Ying Yu, Jun-Li Liu, Dai-Shi Tian

**Affiliations:** 1 Department of Neurology, Tongji Hospital, Tongji Medical College, Huazhong University of Science and Technology, Wuhan, China; 2 Cancer Center, Union Hospital, Tongji Medical College, Huazhong University of Science and Technology, Wuhan, China

**Keywords:** Anti-NMDAR encephalitis, Autoimmune encephalitis, Paraneoplastic limbic encephalitis, Chronic myelogenous leukemia, Seizure, Case report

## Abstract

**Background:**

Anti-N-methyl-D-aspartate receptor (NMDAR) encephalitis is the most frequent autoimmune paraneoplastic encephalitis, and is primarily associated with ovarian teratomas. Here, we report the first case of a patient diagnosed with chronic myelogenous leukemia (CML) during the recovery phase of anti-NMDAR encephalitis.

**Case presentation:**

The patient was admitted with fever, headache, and seizures. Brain MRI revealed a cerebrospinal fluid (CSF)-containing arachnoid cyst in the left temporal lobe with no other abnormal signals. EEG showed diffuse background slowing in the delta-theta range. The patient tested positive for anti-NMDAR antibodies in both the serum and CSF. One year after the onset of encephalitis, the patient was referred to the Department of Hematology for extreme leukocytosis. Karyotype analysis showed the presence of Philadelphia chromosome t(9;22)(q34;q11). Quantitative reverse transcriptase PCR analysis further identified BCR/ABL1 fusion transcripts; thus, CML was diagnosed.

**Conclusions:**

To the best of our knowledge, this is the first case of anti-NMDAR encephalitis associated with CML. This report should alert clinicians to consider CML as a malignancy that is possibly associated with limbic encephalitis.

## Background

Anti-N-methyl-D-aspartate receptor (NMDAR) encephalitis is a type of autoimmune limbic encephalitis characterized by a variety of symptoms, including memory loss, seizures, movement abnormalities, autonomic instability, paranoia, delusions, and catatonia. Dalmau et al. first identified in 2007 that anti-NMDAR encephalitis was caused by autoantibodies targeting the NMDA receptor in the brain [1, 2]. Anti-NMDAR encephalitis can present as an independent non-paraneoplastic disorder, or a paraneoplastic syndrome [3]. As the most frequent autoimmune encephalitis, anti-NMDAR encephalitis is reported to be associated with ovarian teratomas and other malignancies [2, 4–6]. Chronic myelogenous leukemia (CML) is a malignancy of the myeloid cell lineage genetically characterized by the Philadelphia (Ph) chromosome [t(9;22)(q34;q11)], which generates the BCR-ABL1 fusion gene [7]. Here, we report the first case of a patient who was diagnosed with CML during the recovery phase from anti-NMDAR encephalitis.

## Case presentation

Here we present the case of a previously healthy, right-handed 23-year-old man. The chief complaints were fever and headache for two days, accompanied by vomiting. He developed one episode of generalized tonic–clonic seizures (GTCS), for which he was admitted to our hospital.

On admission, he further exhibited focal seizures, anxiety symptoms, sweating, sleep disturbance, and amnesia. He was conscious and oriented toward time, place, and person. His vital signs were within the normal limits. Examination revealed neck stiffness, and his neurological status was normal. A general medical examination revealed no abnormal findings.

Lumbar puncture was performed after admission. The cerebrospinal fluid (CSF) pressure was 220 mmH_2_O. Bacterial, tuberculosis, and fungal cultures were negative. IgM antibodies for cytomegalovirus, rubella virus, herpes simplex virus, parvovirus B19, Epstein-Barr virus, enteroviruses, varicella-zoster virus, and mumps virus were negative. The oligoclonal band was negative in both the CSF and serum. The CSF nucleated cell count was 10 × 10^6^ cells/L (0–8 × 10^6^/L). The red cell count was 3200 × 10^6^ cells/L (< 0/L). The total protein level was 181 mg/L (150–450 mg/L) and the albumin level was 95 mg/L (100–300 mg/L). Glucose, electrolyte, and LDH levels were within the normal limits.

Extensive laboratory evaluation revealed an elevated white blood cell count of 16.31 × 10^9^/L (3.5–9.5 × 10^9^/L), myoglobin count of 160.9 ng/mL (< 154.9 ng/mL), creatine kinase level of 3447 U/L (< 190U/L) and C-reactive protein level of 10.5 mg/L (< 1 mg/L). The following tests showed no abnormalities: hemoglobin, platelet count, D-D dimer, erythrocyte sedimentation rate, thyroid stimulating hormone, free T3, free T4, antinuclear antibody, antineutrophil cytoplasmic antibodies panel, rheumatoid factor, hepatitis B virus, hepatitis C virus, human immunodeficiency virus, and syphilis.

**Fig. 1:**
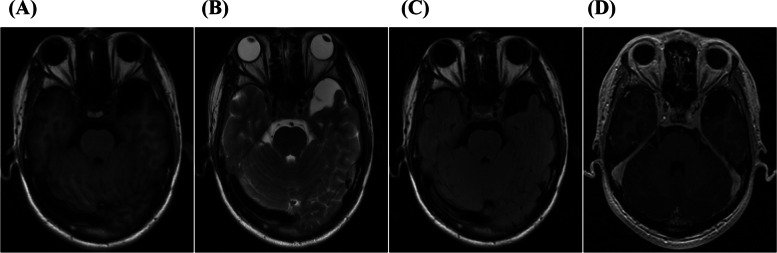
(**A**), (**B**), (**C**), (**D**) represent T1WI, T2WI, FLAIR, and post contrast 3D-BRAVO images. The MRI imaging of his brain was normal except for a CSF-containing arachnoid cyst on the left temporal lobe

Brain magnetic resonance imaging (MRI), including fluid-attenuated inversion recovery sequence and enhanced scanning, revealed a CSF-containing arachnoid cyst in the left temporal lobe, while no other abnormal signals were observed (Fig. 1). EEG showed a diffuse background slowing in the delta-theta range. Chest CT did not reveal any abnormalities. Ultrasound examinations of the heart, liver, gallbladder, spleen, pancreas, kidneys, ureters, bladder, and testis were normal.

A profile of autoimmune encephalitis was obtained. The patient tested positive for anti-NMDAR antibodies both in serum and the CSF (titer 1:100 and 1:32, respectively; using a cell-based immunofluorescence assay), and a diagnosis of anti-NMDAR encephalitis was considered.

He received high-dose intravenous corticosteroids, followed by intravenous immunoglobulin, after which symptoms, such as fever and insomnia, started to improve. Oral corticosteroids were subsequently introduced and gradually reduced; however, severe attention disturbance and amnesia persisted, and tacrolimus was added. Four months after the initial treatment, the patient was almost completely symptom-free. During the visit, his blood tacrolimus fluctuated from 4.1 to 11.37 ng/mL, and the white blood cell count ranged from 8.11 to 12.73 × 10^9^/L (3.5–9.5 × 10^9^/L). However, 1 year after the onset of anti-NMDAR encephalitis, routine blood examination revealed a markedly elevated white blood cell count of 119.18 × 10^9^/L (3.5–9.5 × 10^9^/L), confirmed on repeat test to be 124.69 × 10^9^/L. He was then referred to our department of hematology for evaluation of extreme leukocytosis.

On admission, the patient denied fatigue, weakness, abdominal pain, digital pain, multiple arthralgia, or bleeding. A physical examination performed at the Department of Hematology revealed no abnormalities. The patient's abdomen was not distended, and the spleen was not palpable.

Besides hyperleukocytosis, the peripheral blood counts were as follows: hemoglobin level 151 g/L (130–175 g/L), platelets 166.0 × 10^9^/L (125–350 × 10^9^/L). Laboratory analyses of the blood were normal for ANA, ANCA, erythrocyte sedimentation rate, creatinine, urea, alanine aminotransferase, aspartate aminotransferase, bilirubin, and total proteins. Serum ferritin levels were high at 1880.9 ng/mL (30–400 ng/mL), and lactate dehydrogenase was 1740 U/L (135–225 U/L). C-reactive protein level was positive at 5.2 mg/L (< 1 mg/L).

**Fig. 2:**
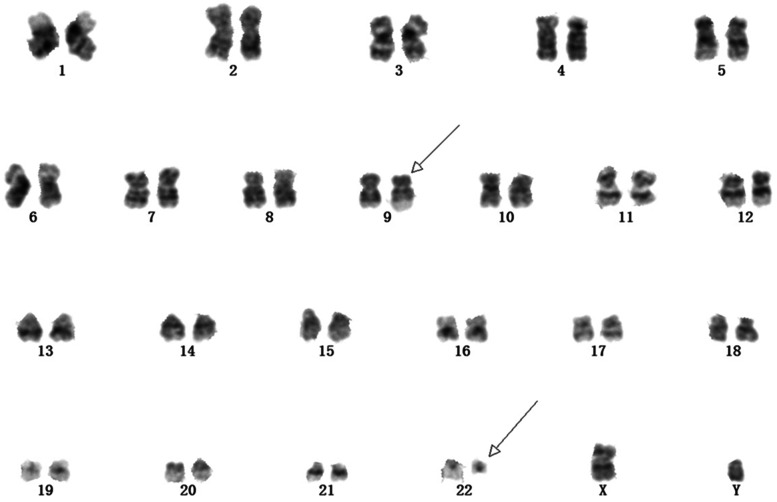
The karyotype from conventional cytogenetic analysis shows the t(9;22)(q34;q11) (arrows)

Six metaphases were assessed for karyotype analysis from the bone marrow specimen, all of which showed the classic Philadelphia chromosome resulting from t(9;22)(q34;q11) (Fig. 2). Quantitative reverse transcriptase PCR analysis identified BCR/ABL1 fusion transcripts with a predominant p210 transcript at a BCR/ABL ratio of approximately 215.47%. CML was diagnosed, and treatment with hydroxyurea was initiated.

## Discussion and conclusion

To the best of our knowledge, this is the first reported case of anti-NMDAR encephalitis associated with CML. The diagnostic criteria for paraneoplastic limbic encephalitis (PLE) include cancer that presents within the next 5 years, classical or non-classical neurological syndromes, and positive neuronal antibodies [8]. PLE is most commonly associated with small cell lung carcinomas and testicular germ cell tumors [9]. It may also be due to teratomas, thymomas, bladder cancers, and breast cancers [9]. Regarding hematological malignancies, Hodgkin’s lymphoma is the most common cause of PLE, while T-cell lymphoma, chronic lymphoid leukemia, and acute myelogenous leukemia have also been reported [9–12]. To the best of our knowledge, no case of CML with PLE has been reported to date.

Anti-NMDAR encephalitis is a form of limbic encephalitis that can be either paraneoplastic or nonparaneoplastic [13]. The NMDA receptor is a glutamate receptor primarily expressed in the cerebral cortex and hypothalamus [14]. As the most abundant excitatory neurotransmitter in the brain, glutamate is involved in cognitive functions such as learning and memory [15]. Infection, inflammation, and neoplasms can initiate molecular mimicry of the NMDA receptor, which in turn results in a mistaken attack on the healthy brain tissue [16–18]. Studies have suggested that viral infection can induce neuroinflammation, leading to anti-NMDAR antibody production [19]. Herpes simplex is the most commonly identified trigger of the encephalitic autoimmune response, and several studies have reported associations between anti-NMDAR encephalitis and other viruses or bacteria [20]. Despite extensive testing, no specific cause of antibody formation after infection has been identified. Approximately 20–40% of patients with anti-NMDAR encephalitis have an associated tumor, and the main tumor type is ovarian teratoma, which expresses NMDAR [21–23]. Testicular immature teratoma and lymphoma are also found to be associated with anti-NMDAR encephalitis [21, 24]. However, further investigation is required to determine the cause of NMDAR autoantibody formation and its correlation with malignancy.

The pathogenesis of CML is multifactorial, and genetic, metabolic, and immunological factors appear to play critical roles [25]. Autoimmune diseases are mainly characterized by T cell lymphocytes reactive with host antigens or B cell-forming autoantibodies against host antigens [16]. Studies have demonstrated that many autoimmune diseases are associated with CML, including Guillain–Barré syndrome, chronic inflammatory demyelinating polyneuropathy, multiple sclerosis and myasthenia gravis [26, 27]. Regarding the mechanisms involved, abnormally proliferating monocytes and further dysregulation of the T cell population are assumed to play an important role [28]. Further studies are required to explore the exact role of monocytes in patients with coexisting anti-NMDAR encephalitis and CML. In addition, accumulating evidence has suggested that autoimmune diseases might trigger hematological malignancies due to a common genetic predisposition. For example, human leukocyte antigen (HLA-B27) is associated with the etiology of autoimmune diseases such as ankylosing spondylitis and amyloidosis, and recent studies have suggested that HLA-B27 carriers may be predisposed to lymphoid malignancies [29, 30]. It is therefore possible that such a link may exist between anti-NMDAR encephalitis and CML. However, further studies are required to clarify this point.

Overall, we report the first case of a patient diagnosed with CML one year after the onset of anti-NMDAR encephalitis. It is still unclear as to whether CML is correlated with anti-NMDAR encephalitis, or developed simply by coincidence. Although more cases and studies are required to support a causal relationship, it may be prudent to consider the alternative explanation that the patient coincidentally developed anti-NMDAR encephalitis of unknown cause and CML within five years of disease symptom onset. Tacrolimus is a calcineurin inhibitor that can be used as a graft-versus-host disease prophylaxis following allogeneic hematopoietic cell transplantation in hematological malignancies, such as CML. This treatment exerts adverse effects on the hematological system, including anemia, leukopenia, leukocytosis, and thrombocytopenia [31, 32]. To date, no CML attributed to tacrolimus has yet been reported. This report should alert clinicians to consider CML as a malignancy potentially associated with limbic encephalitis. For patients diagnosed with anti-NMDAR encephalitis, continuous routine blood tests should also be recommended. We look forward to developing studies to answer important questions regarding the mechanisms involved.

## Data Availability

The datasets used and/or analysed during the current study are available from the corresponding author on reasonable request.

## Abbreviations

NMDAR: N-methyl-D-aspartate receptor
CSF: Cerebrospinal fluid
PCR: Polymerase chain reaction
PLE: Paraneoplastic limbic encephalitis
CML: Chronic myelogenous leukemia
Ph: Philadelphia
GTCS: Generalised tonic clonic seizures
MRI: Magnetic resonance imaging
EEG: Electroencephalogram
ANA: Antinuclear antibodies
ANCA: Anti-Neutrophil cytoplasm autoantibodies
HLA: Human leucocyte antigen
